# A Shade Intensity Threshold Triggers Gibberellin Metabolic Reprogramming to Drive Shade Avoidance in Soybean

**DOI:** 10.3390/ijms27156896

**Published:** 2026-08-01

**Authors:** Lin Wang, Suya Qiu, Xiao Xu, Ruineng Xu, Hong Liao, Yongdong Yu

**Affiliations:** Root Biology Center, Fujian Agriculture and Forestry University, Fuzhou 350002, China; 17860827998@163.com (L.W.); qsy13228141719@163.com (S.Q.); 19847567618@163.com (X.X.); ruinengxu@163.com (R.X.); hliao@fafu.edu.cn (H.L.)

**Keywords:** shade, soybean, plant height, gibberellin

## Abstract

Shade stress severely constrains soybean yield in soybean-maize intercropping systems, yet the intensity threshold triggering shade avoidance responses and the underlying hormonal mechanisms remain elusive. Here, through two-year field experiments, we demonstrate that shading coverage rate exceeding 80% is the critical threshold initiating shade avoidance, increasing plant height by 16–61%. The seventh internode is the initial responsive site where epidermal cells elongate by 69.86% longitudinally, while radial growth is broadly suppressed (cell area reduced by approximately 41%). Furthermore, integrated hormonal profiling and gene expression analyses demonstrate that shade promotes active gibberellin (GA) accumulation via dual metabolic reprogramming: upregulating biosynthetic genes *GmGA20ox1* and *GmGA3ox1* and downregulating catabolic genes *GmGA2ox*–*7a* and *GmGA2ox*–*7b*, leading to a 56.87% increase in GA_1_ content. Notably, exogenous GA fully mimics the shade-induced phenotype, with genotype sensitivity ranking BX10 > BD2 > W82, indicating that GA plays a central role in mediating the shade response. Yield analysis shows that shade inhibits soybean biomass accumulation and grain yield and preferentially suppresses reproductive rather than vegetative growth, reducing effective pod number and seeds per plant by ~50% whereas 100-seed weight only decreases by 6.55%. Together, these findings reveal that shade modulates GA homeostasis via “enhanced biosynthesis and suppressed catabolism” to remodel internodes, providing a theoretical basis for shade-tolerant soybean breeding and optimizing intercropping systems.

## 1. Introduction

Soybean (*Glycine max* [L.] Merr.) serves as a strategic crop for grain, oil, and feed uses, playing a pivotal role in global food security and sustainable agriculture [1,2,3,4]. With growing global populations and rising living standards, soybean demand has steadily increased. However, traditional monoculture systems face mounting pressures from limited arable land and stagnating yield, driving the adoption of maize–soybean strip intercropping [5]. This system leverages niche complementarity between the two species to enhance land–use efficiency, improve soil health through biological nitrogen fixation, and reduce pest and disease incidence [6,7,8]. It has been widely promoted in China and other regions as a sustainable approach to meeting rising soybean demand without expanding cultivated area [9].

Despite these agronomic advantages, intercropping imposes a major challenge on the lower–canopy soybean. Maize plants are tall with broad leaves and dense canopies, casting continuous shade on adjacent soybean rows during middle and late growth stages [10,11]. Light is a critical environmental factor for soybean growth, directly regulating photosynthesis, plant architectural, and yield formation [12,13,14,15]. Shading triggers a typical shade-avoidance syndrome (SAS), characterized by rapid stem elongation, increased internode length, and reduced stem diameter, collectively resulting in a slender morphology, elevated lodging risk, and ultimately yield penalties [16,17]. These morphological adaptations reflect an evolutionary trade–off: enhanced light capture at the expense of mechanical stability and reproductive allocation [18].

The molecular mechanisms underlying shade–induced elongation have been extensively characterized in *Arabidopsis thaliana*, where low red–to–far–red (R/FR) ratios activate phytochrome–interacting factors (PIFs) to promote auxin and gibberellin (GA) biosynthesis, driving cell elongation [19,20,21,22,23]. However, the regulatory architecture in soybean remains poorly understood. Recent studies have identified key components of the shade–GA signaling axis: the cryptochrome GmCRY1s mediates weak blue–light perception; its down-regulation under shade reduces GA oxidase expression, thereby increasing GA content and promoting stem elongation [24]. Additionally, the E3 ubiquitin ligase GmILPA1 regulates GA catabolism by interacting with GA2ox proteins to maintain GA homeostasis [25]. Nevertheless, these findings focus primarily on upstream photoreceptor signaling or post-translational regulation. A critical gap remains regarding how shade dynamically reprograms GA metabolic flux at the transcriptional level to coordinate internode–specific elongation.

Gibberellins are central regulators of plant cell elongation, with active forms—particularly GA_1_—promoting stem growth through cell wall loosening and turgor–driven expansion [26,27,28,29,30]. The GA metabolic network is precisely controlled by opposing biosynthetic and catabolic enzymes: GA20ox and GA3ox catalyze the production of bioactive GAs, whereas *GA2ox* family members mediate their inactivation [31,32,33,34]. In shade–avoiding species, rapid adjustments in the balance between these enzymatic activities enable swift hormonal responses to fluctuating light environments [35]. Nevertheless, whether a critical shading intensity threshold exists, which specific internodes respond first, and how shade orchestrates a coordinated metabolic reprogramming that simultaneously enhances GA biosynthesis and suppresses GA catabolism to drive anisotropic growth have not been systematically addressed in soybean.

Despite previous reports on shade-induced GA changes, the critical shade intensity threshold, the specific internodal response pattern, and the underlying transcriptional regulatory mechanism of GA metabolic reprogramming remain largely uncharacterized in field-grown soybean. To address these gaps, we combined two-year field experiments with controlled indoor assays to systematically investigate the dynamic architectural remodeling of soybean under shading. By integrating phenotyping, cytology, hormone profiling, gene expression analysis, and exogenous GA_3_ application, we show that shading coverage >80% triggers a dual “enhanced biosynthesis-suppressed catabolism” reprogramming of GA_1_ metabolism, which drives anisotropic cell elongation at specific internodes and leads to characteristic architectural changes. These findings not only advance our mechanistic understanding of soybean shade avoidance at the hormonal and transcriptional levels, but also provide candidate molecular targets for breeding shade-tolerant, lodging-resistant soybean varieties and optimizing maize-soybean intercropping systems.

## 2. Results

### 2.1. Shade Dynamically Increases Soybean Plant Height but Inhibits Stem Diameter

**Figure 1:**
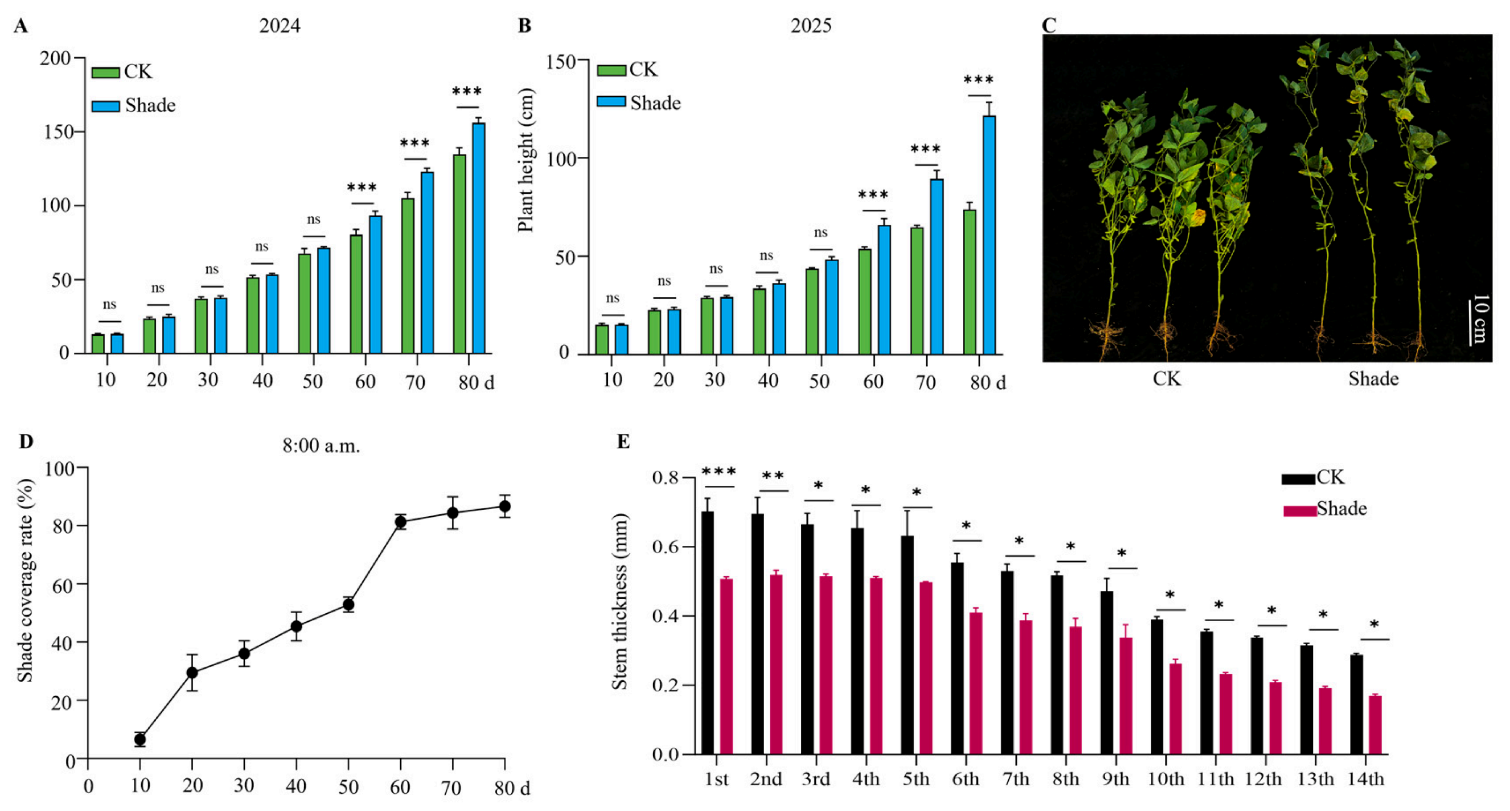
Effects of shading on plant height and stem diameter of soybean. (**A**) Plant height dynamics of soybean from sowing to 80 days under shading and control (CK) conditions in 2024. (**B**) Plant height dynamics of soybean from sowing to 80 days under shading and CK conditions in 2025. (**C**) Field phenotype of 60–day–old soybean plants in 2025, Bar = 10 cm. (**D**) Shading percentage imposed on intercropped soybean by associated maize in 2025 field conditions, measured at 8:00 a.m. (**E**) Internode–specific stem diameter variations in 80–day–old soybean under shading and CK conditions in 2025. d, days after sowing. Data in (**A**,**B**,**E**) represent means ± SD (*n* = 8) and asterisks indicate significant differences (* *p* < 0.05, ** *p* < 0.01, and *** *p* < 0.001). A two-tailed Student’s *t*-test was used to determine *p* values. ns, not significant.

To elucidate the dynamic regulatory mechanism of maize shading on intercropped soybean growth, we conducted two–year field experiments in 2024 and 2025. No significant difference in plant height was observed between shaded and control plants from 10 to 50 days after sowing (DAS) (Figure 1A,B). However, shaded plants exhibited significantly greater height than controls starting at 60 DAS, and this difference increased progressively with plant development (Figure 1A–C). In 2024, shading increased plant height by 16.05%, 16.94%, and 17.28% at 60, 70, and 80 DAS, respectively; in 2025, the increases were more pronounced, reaching 23.09%, 39.03%, and 61.32% (Figure 1A,B). This temporal pattern was highly correlated with changes in maize canopy shading intensity which rose from merely 7% at 10 DAS to 85% at 80 DAS based on measurements taken at 8:00 a.m. (Figure 1D). Stem diameter analysis showed that shading severely suppressed radial growth. At the R6 stage (80 DAS) in 2025, stem diameters of all nodes from the 1st to the 14th were significantly reduced by 20–50% compared to the control (Figure 1E). These results demonstrate that shading coverage exceeding 80% represents the critical threshold triggering soybean shade avoidance syndrome, characterized by rapid internode elongation and reduced stem diameter.

### 2.2. Shade Specifically Promotes Elongation of the Seventh and Upper Internodes

**Figure 2:**
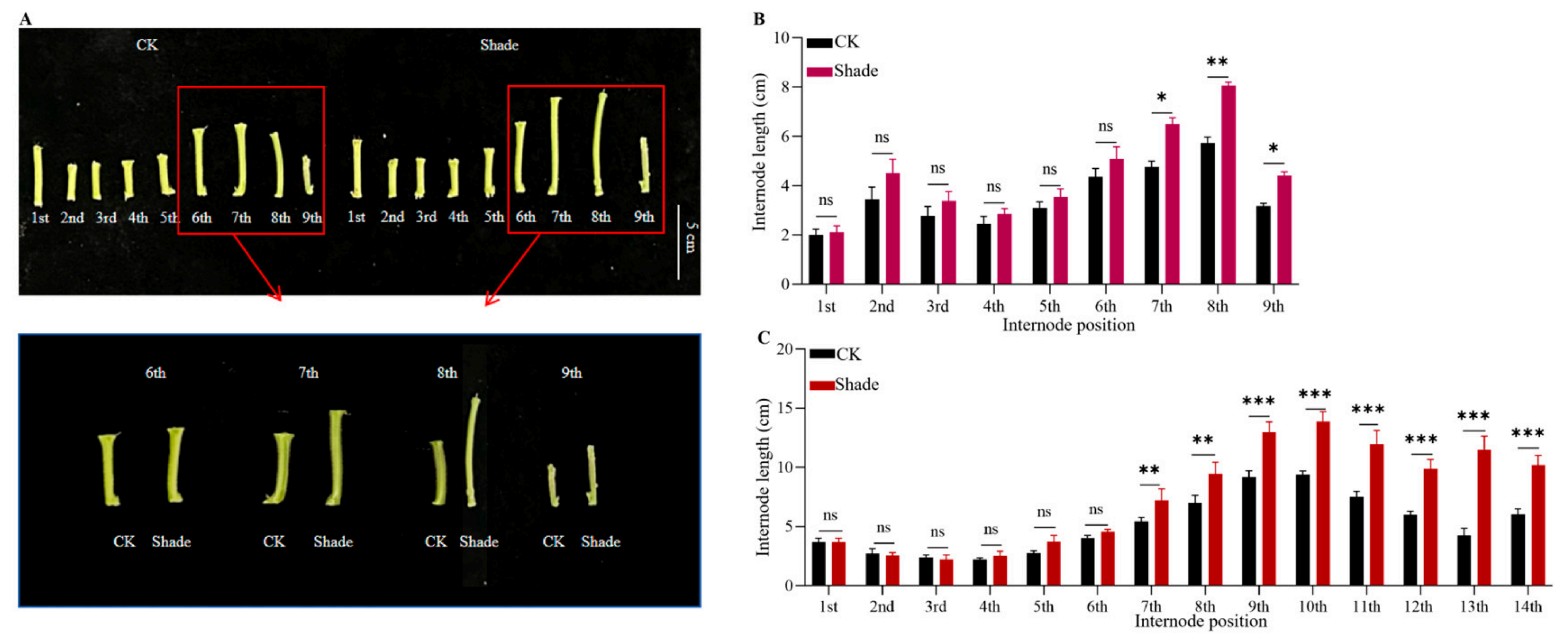
Effects of shading on the soybean node spacing. (**A**) Internodes of 60–day–old soybean plants under shading and CK conditions in 2025, Bar = 5 cm. (**B**) Internode length of 60–day–old soybean plants under shading and CK conditions in 2025. (**C**) Internode length of 80–day–old soybean plants under shading and CK conditions in 2025. Data in (**B**,**C**) represent means ± SD (*n* = 8) and asterisks indicate significant differences (* *p* < 0.05, ** *p* < 0.01, and *** *p* < 0.001). A two-tailed Student’s *t*-test was used to determine *p* values. ns, not significant.

To investigate the node-specific pattern of shade-induced plant height increase, we systematically measured dynamic changes in internode length across different nodes. At 60 DAS, the first significant difference between shaded and control plants appeared at the seventh internode, where shading increased length by 37.65%; no significant differences were detected in internodes 1–6 (Figure 2A,B). By 80 DAS, all internodes from the seventh upward were significantly longer in shaded plants (Figure 2C). These results indicate that the seventh internode is the initial site of shade response, and the effect propagates acropetally and intensifies with plant development.

### 2.3. Shade Induces Anisotropic Growth of Soybean Stem Cells

**Figure 3:**
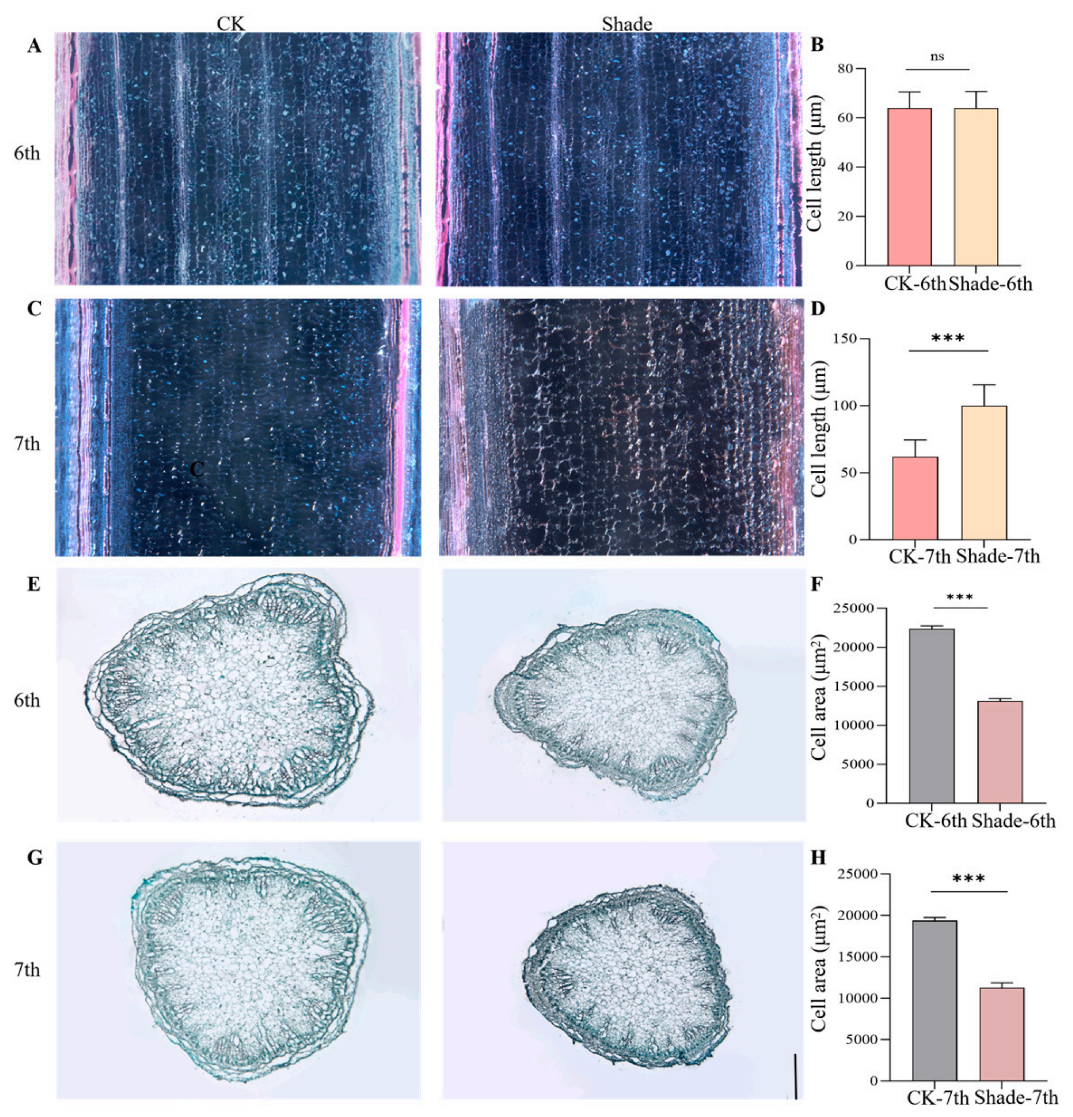
Effects of shading on internode cell length of soybean. (**A**) Longitudinal section phenotypes of internode cells in the sixth internodes of soybean under shading and CK conditions in the soybean–maize intercropping system, Bar = 500 μm. (**B**) Internode cell length of the sixth internodes of soybean under shading and CK conditions in the soybean–maize intercropping system. (**C**) Longitudinal section phenotypes of internode cells in the seventh internodes of soybean under shading and CK conditions in the soybean–maize intercropping system, Bar = 500 μm. (**D**) Internode cell length of the seventh internodes of soybean under shading and CK conditions in the soybean–maize intercropping system. (**E**) Transverse section phenotypes of internode cells in the sixth internodes of soybean under shading and CK conditions in the soybean–maize intercropping system, Bar = 500 μm. (**F**) Cross–sectional cell area of the sixth internodes of soybean under shading and CK conditions in the soybean–maize intercropping system. (**G**) Transverse section phenotypes of internode cells in the seventh internodes of soybean under shading and CK conditions in the soybean–maize intercropping system, Bar = 500 μm. (**H**) Cross–sectional cell area of the seventh internodes of soybean under shading and CK conditions in the soybean–maize intercropping system. Data in (**B**,**D**,**F**,**H**) represent means ± SD (*n* = 8) and asterisks indicate significant differences (*** *p* < 0.001). A two-tailed Student’s *t*-test was used to determine *p* values. ns, not significant.

To verify the direct mechanism of internode elongation at the cellular level, we performed microscopic analysis of sixth and seventh internodes. Longitudinal sections revealed that epidermal cell length in the seventh internode increased by 69.86% under shade, whereas no change was detected in the sixth internode (Figure 3A–D), confirming that rapid longitudinal elongation of epidermal cells directly drives internode extension. In contrast, cross-section analysis showed that stem cell areas in both the sixth and seventh internodes of shade plants were significantly reduced by 41.37% and 41.79%, respectively (Figure 3F or H). This radial suppression was consistently observed in cortical, phloem, xylem, and pith cells (Figure 3E or G). Taken together, shade drives the formation of a slender stem phenotype through an “anisotropic growth” strategy: promoting longitudinal elongation of epidermal cells at specific nodes while comprehensively inhibiting radial growth of all cell types.

### 2.4. Dose-Dependent Effect of Light Intensity on Soybean Architecture

**Figure 4:**
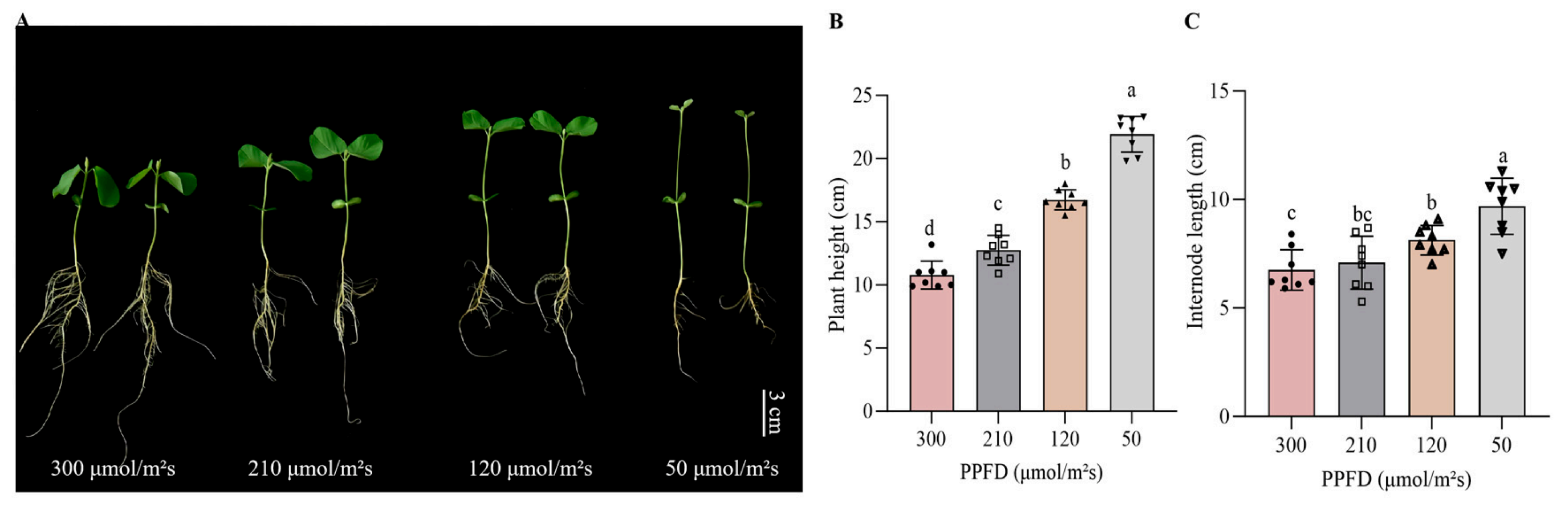
Effects of different light intensities on the growth of soybean. (**A**) Phenotypes of soybean under different gradient light intensities, Bar = 3 cm. (**B**) Plant height of soybean under different light intensities. (**C**) Hypocotyl length of soybean under different light intensities. Bar graphs labeled with different letters represent statistically significant differences, as determined by one-way analysis of variance (ANOVA) with Tukey’s test.

To verify the dose-dependent effect of light intensity on soybean architecture, we exposed plants to four photosynthetic photon flux density (PPFD) gradients: 300, 210, 120, and 50 μmol·m^−2^·s^−1^ under controlled indoor conditions. After 15 days of treatment, soybean plant height and hypocotyl length increased significantly as light intensity decreased (Figure 4A). Notably, plants under 50 μmol·m^−2^·s^−1^ exhibited significantly greater plant height and hypocotyl length than all other treatment groups, displaying a typical shade avoidance phenotype (Figure 4B,C). These results confirm that light intensity serves as the direct environmental signal regulating soybean shade avoidance, with response magnitude positively correlated with shading severity.

### 2.5. Shade Reprograms GA Metabolism to Promote Active Hormone Accumulation

**Figure 5:**
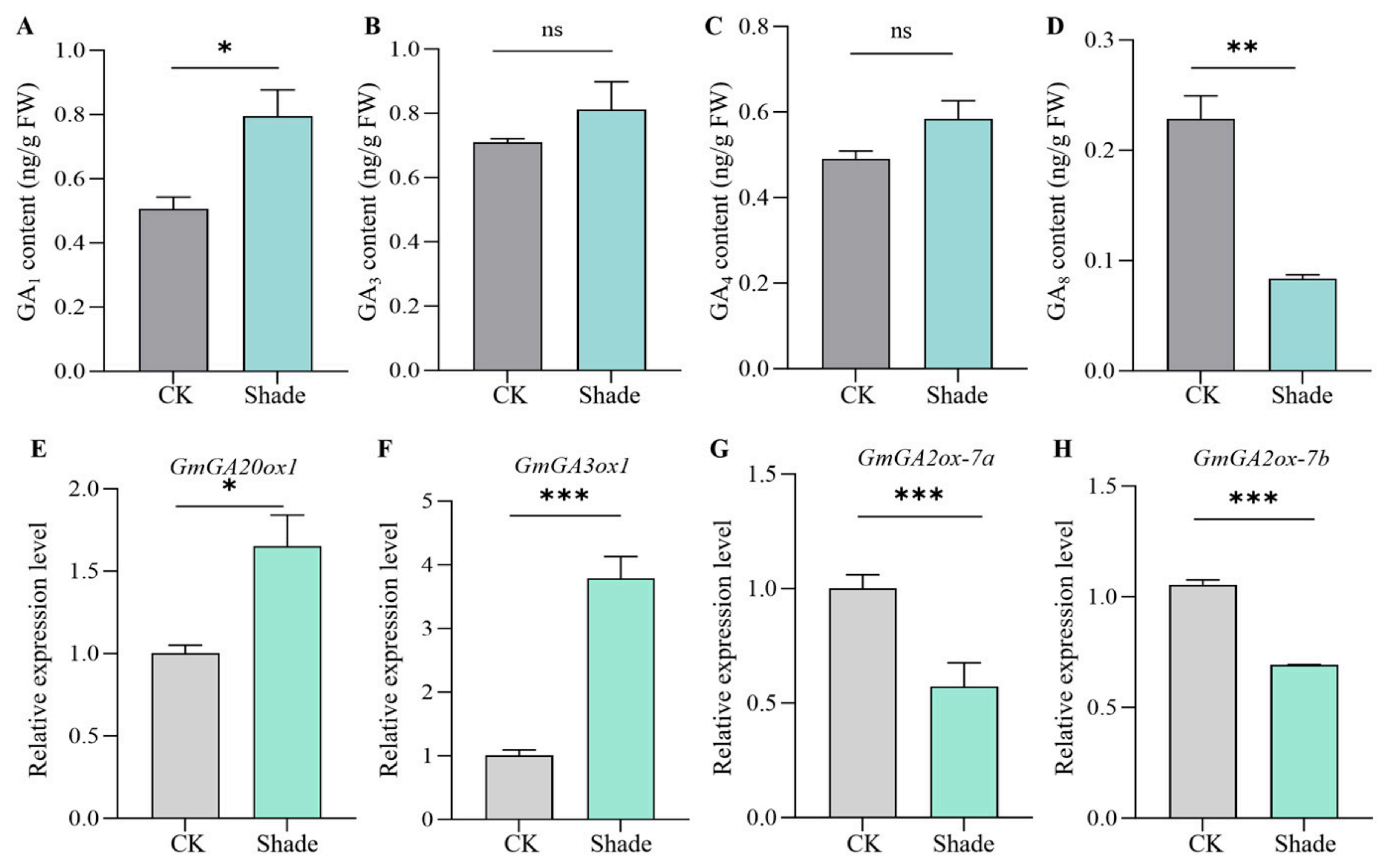
Effects of shading on gibberellin in soybean internodes. (**A**) GA_1_ content in the seventh internode of 60–day–old soybean under shading and CK conditions. (**B**) GA_3_ content. (**C**) GA_4_ content. (**D**) GA_8_ content. (**E**) Expression analysis of *GmGA20ox1* in the seventh internode of 60–day–old soybean under shading and CK conditions. (**F**) Expression analysis of *GmGA3ox1*. (**G**) Expression analysis of *GmGA2ox*–*7a*. (**H**) Expression analysis of *GmGA2ox*–*7b*. Data represent means ± SD (*n* = 8) and asterisks indicate significant differences (* *p* < 0.05, ** *p* < 0.01, and *** *p* < 0.001). A two-tailed Student’s *t*-test was used to determine *p* values. ns, not significant.

To dissect the hormonal mechanism underlying shade-regulated internode elongation, we profiled gibberellin (GA) metabolites in the seventh internode. Compared to the control, shade significantly increased the content of bioactive GA_1_ by 56.87% (Figure 5A), while GA_3_ and GA_4_ showed upward trends that did not reach statistical significance (Figure 5B,C). In contrast, the content of GA_8_, the main inactive catabolite of GA_1_, decreased by 63.42% (Figure 5D). Quantitative real-time PCR analysis revealed that shade significantly upregulated expression of GA_1_ biosynthetic genes: *GmGA20ox1* by 64.67% and *GmGA3ox1* by 275.34% (Figure 5E,F). Concurrently, shade downregulated GA_1_ catabolic genes: *GmGA2ox*–*7a* by 42.86% and *GmGA2ox*–*7b* by 34.39% (Figure 5G,H). These findings demonstrate that shade promotes active GA_1_ accumulation through dual transcriptional reprogramming, namely enhanced biosynthesis coupled with suppressed catabolism, which in turn drives internode cell elongation.

### 2.6. Exogenous GA Mimics Shade Phenotypes and Reveals Genotype-Specific Sensitivity

**Figure 6:**
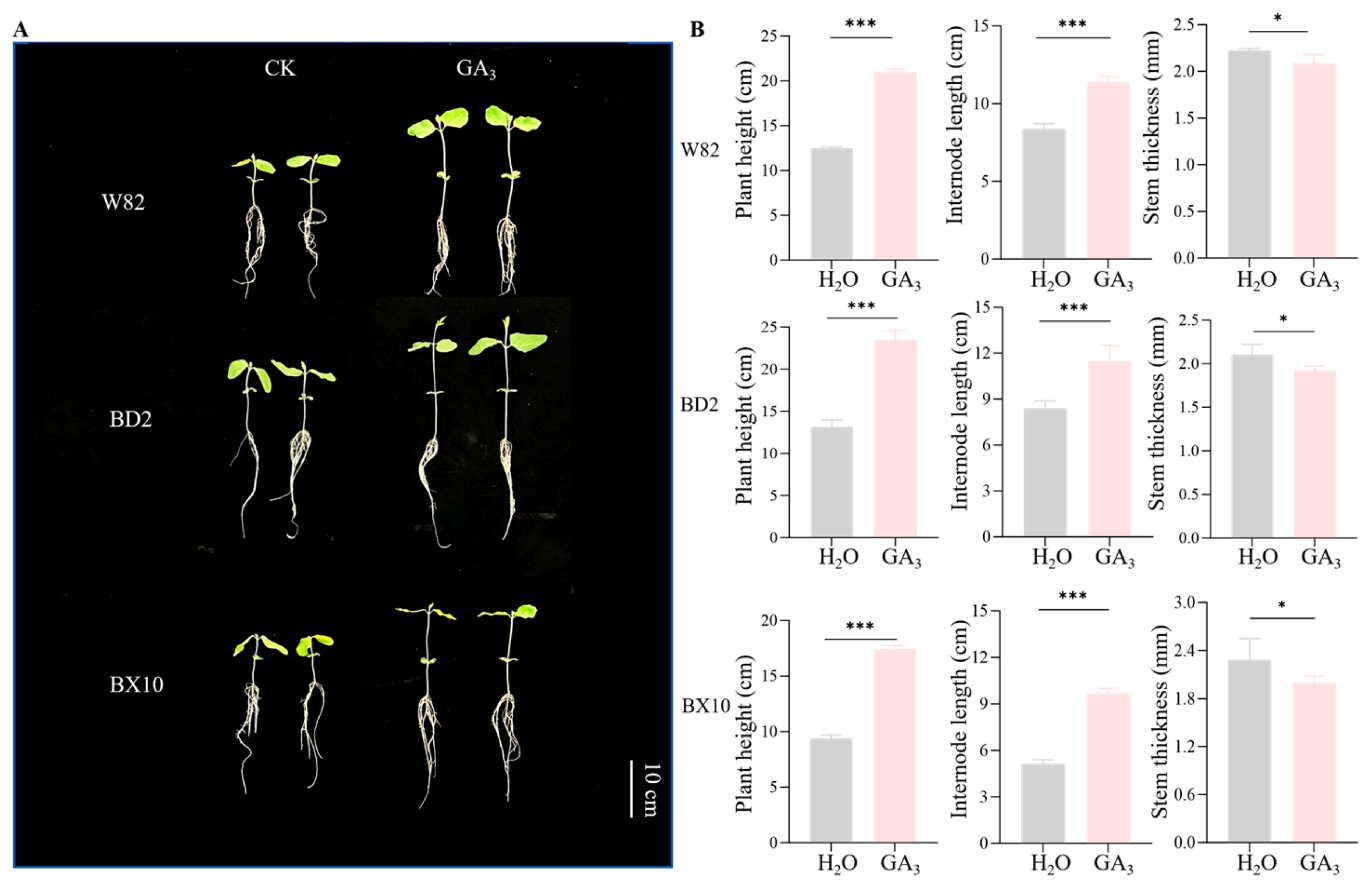
Effects of exogenous GA_3_ application on soybean growth. (**A**) Phenotypes of soybean cultivars W82, BX10, and BD2 at 20 days after foliar application of water and 10 μM GA_3_ in hydroponic culture, Bar = 10 cm. (**B**) Quantitative measurements of plant height, internode length, and stem diameter of soybean cultivars W82, BX10, and BD2 at 20 days after foliar application of water and 10 μM GA_3_ in hydroponic culture. Data in (**B**) represent means ± SD (*n* = 8) and asterisks indicate significant differences (* *p* < 0.05, *** *p* < 0.001). A two-tailed Student’s *t*-test was used to determine *p* values.

To functionally validate the role of gibberellins in shade-induced soybean architecture, in this study, three genotypes (W82, BX10, BD2) were foliar-sprayed with 10 μM GA_3_, with water as control. Agronomic traits including plant height, internode length, and stem diameter were measured 20 days after sowing. GA_3_ treatment significantly increased plant height and internode length in all three genotypes, with marked genotypic differences in sensitivity (Figure 6A). BX10 showed the highest increases (85.71% in height, 89.45% in internode length), whereas W82 exhibited the smallest responses. Conversely, GA_3_ severely suppressed stem diameter in all genotypes, with BX10 showing the greatest reduction (12.61%) and W82 the least (6.28%) (Figure 6B). These results indicate that exogenous GA_3_ fully recapitulates shade-induced architectural changes, specifically promoting elongation and suppressing thickening, and that intrinsic genetic variation in GA sensitivity exists among soybean genotypes. This further confirms GA as a key signaling molecule in shade-induced internode elongation, and the differential sensitivity provides a physiological marker for breeding programs.

### 2.7. Shade Severely Inhibits Soybean Biomass Accumulation and Grain Yield

**Figure 7:**
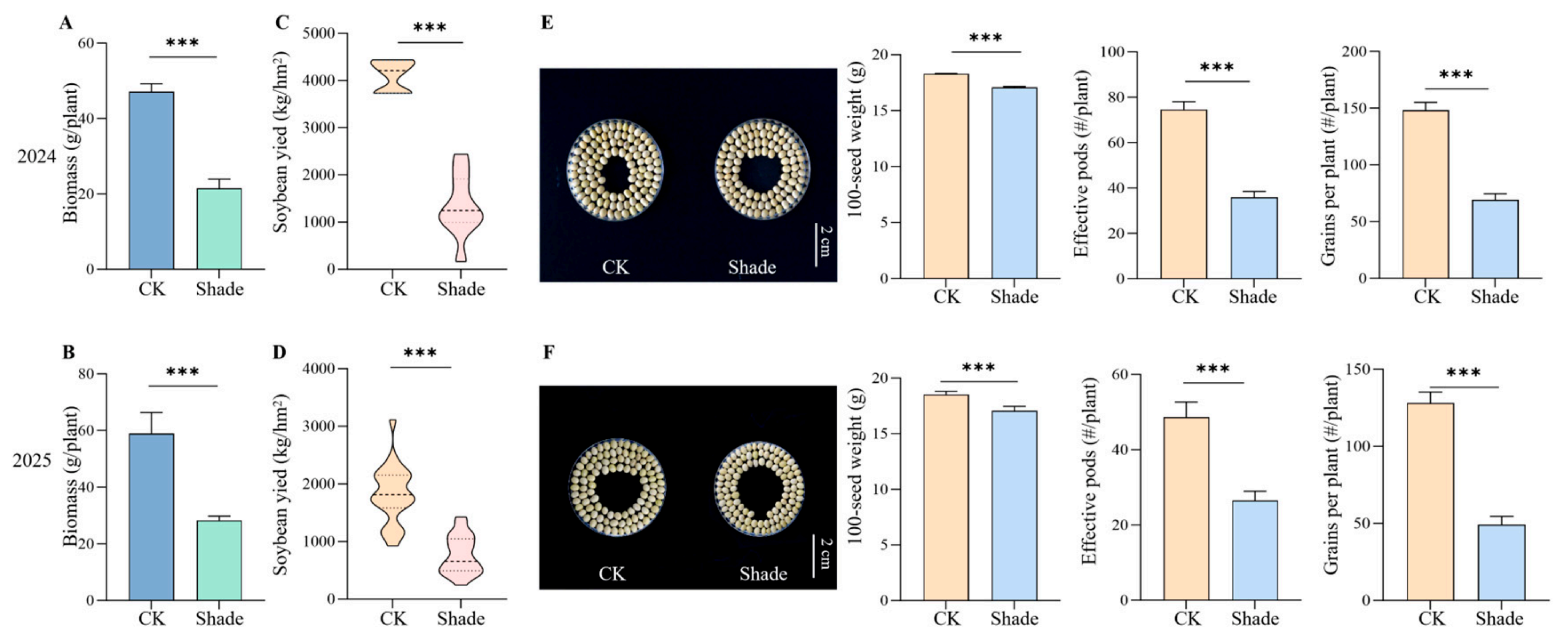
Effects of shading on the soybean yield. (**A**) Biomass of soybean under shading and CK conditions in 2024. (**B**) Biomass of soybean under shading and CK conditions in 2025. (**C**) Yield of soybean under shading and CK conditions in 2024. (**D**) Yield of soybean under shading and CK conditions in 2025. (**E**) Yield components of soybean under shading and CK conditions in 2024, Bar = 2 cm. (**F**) Yield components of soybean under shading and CK conditions in 2025, Bar = 2 cm. Data represent means ± SD (*n* = 8) and asterisks indicate significant differences (*** *p* < 0.001). A two-tailed Student’s *t*-test was used to determine *p* values.

To assess the impact of shade on soybean yield and to dissect the key factors limiting productivity, we measured biomass and yield components in the two–year field experiments. Shading reduced aboveground biomass by 54.31% (2024) and 52.07% (2025) (Figure 7A,B), and grain yield by 67.54% (2024) and 57.85% (2025) (Figure 7C,D). Analysis of yield components revealed that 100-seed weight decreased only modestly (6.55% in 2024, 7.90% in 2025), whereas effective pod number dropped by 51.89% and 45.55%, and seeds per plant by 53.22% and 61.53%, respectively (Figure 7E,F). The much larger reductions in effective pod number and seeds per plant compared to seed weight indicate that shade impairs reproductive growth far more severely than vegetative growth. Increased flower and pod abscission, leading to reduced pod set, is the primary cause of yield loss under shading.

## 3. Discussion

Shade stress represents a major environmental constraint limiting crop productivity in intercropping systems worldwide [36]. In soybean–maize intercropping, the progressive development of maize canopies creates a dynamic shading environment that triggers dramatic changes in soybean plant architecture, ultimately leading to significant yield losses [37]. While shade avoidance responses have been extensively characterized in model plants, the intensity threshold triggering these responses in field–grown soybean and the underlying hormonal regulatory mechanisms remain poorly understood. In this study, we combined two–year field experiments with controlled indoor assays to systematically investigate the cytological and molecular basis of shading–induced plant architecture remodeling in soybean.

### 3.1. Shade Intensity Threshold Determines Initiation of Shade Avoidance in Soybean

Plant responses to light limitation are highly dose–dependent, with different thresholds triggering distinct adaptive strategies [38]. It is well established that plants can acclimate to mild shading through adjustments in photosynthetic efficiency and leaf orientation without initiating the energetically costly shade avoidance syndrome [37,39]. In this study, a critical threshold of >80% shading coverage was identified as the trigger for significant shade avoidance responses in soybean (Figure 1D). Below this threshold, no significant differences in plant height were observed between shaded and control plants (Figure 1A–C); above it, soybean plants initiated rapid internode elongation accompanied by marked reductions in stem diameter (Figure 1E). This is consistent with studies reporting that shade avoidance responses exhibit intensity dependence in *Arabidopsis*, where low R/FR ratios below 0.5 trigger significant elongation through PIF protein accumulation [40]. However, the present study represents the quantification of this threshold in a field crop and establishes its dynamic association with maize canopy development, providing directly actionable parameters for intercropping system design. While Luo et al. [5] reported that shade–tolerant soybean cultivars mitigate yield losses through optimized canopy architecture, the present findings complement this by revealing the signaling threshold that initiates avoidance responses. The pronounced inter-year variation in height response (16–17% versus 23–61%) likely reflects genotype-by-environment interactions involving cultivar differences, sowing season, temperature and photoperiod. That the core findings—shading threshold, nodal response specificity, and GA_1_ reprogramming—remained consistent across years underscores their robustness, although multi-environment trials with standardized cultivars are needed to partition these independent effects. Though phenotypically robust, the >80% threshold awaits molecular validation. Paired hormone-gene dose-response analyses across the full shading gradient are needed to quantitatively link light intensity to GA metabolic flux.

### 3.2. Node Specificity and Temporal Dynamics of Shade Response

The spatial pattern of internode development is central to plant architectural responses [41,42]. Previous studies have shown that different internodes exhibit distinct sensitivities to environmental signals, with younger, upper internodes generally being more responsive to hormonal and light cues [30]. In this study, shading regulation of soybean internode elongation was found to exhibit striking node specificity and temporal dynamics. Under shade, significant differences between shaded and control plants first emerged at the seventh internode at 60 DAS (Figure 2A,B), extending to all internodes above the seventh by 80 DAS (Figure 2C). This node specificity likely correlates with the spatiotemporal sequence of soybean internode development: lower internodes (1–6) have completed most of their elongation before shading becomes significant, while upper internodes (7 and above) are in their rapid elongation phase and thus more sensitive to hormonal signals [33]. Similar node–specific responses to shading have been reported in other crops such as maize [43] and rice [44], suggesting that this may represent a conserved adaptive strategy across plant species. Cytological analyses further revealed that the increased internode length at the 7th node was due to longitudinal elongation of epidermal cells rather than enhanced cell division, while reduced stem diameter arose from uniform radial growth inhibition across all cell types. This asymmetric growth pattern constitutes the cytological basis for the “slender stem” phenotype and increased lodging risk under shading conditions [45,46,47,48]. Direct biomechanical metrics (e.g., stem breaking force, bending modulus) and cell wall compositional data (e.g., lignin, cellulose content) are lacking in the current study. The observed reductions in cell cross-sectional area (~41%) and stem diameter (20–50%) provide indicative morphological evidence of elevated lodging risk, and future work will integrate these mechanical and biochemical assays for quantitative validation.

### 3.3. GA_1_ Metabolic Reprogramming as the Core Physiological Mechanism of Shade Response

Gibberellins are key phytohormones that regulate plant growth and development, playing a central role in mediating shade avoidance responses [21]. GA_1_, as the major bioactive gibberellin, directly mediates cell elongation by promoting cell wall loosening and turgor-driven expansion [33,49,50]. Shade simultaneously activates biosynthetic genes (upregulation of *GmGA20ox1* and *GmGA3ox1*) (Figure 5E,F) and suppresses catabolic genes (downregulation of *GmGA2ox–7a* and *GmGA2ox–7b*), synergistically promoting bioactive GA_1_ accumulation (Figure 5G,H). This dual regulation mode is more efficient than single–pathway modulation that enables rapid amplification of hormonal signals responses to environmental changes [51]. These results are corroborated by a study showing that GmCRY1s participate in soybean shade avoidance by regulating GA metabolism [24]. GmCRY1s negatively regulate shade avoidance through stabilizing DELLA proteins to suppress GA signaling; in contrast, the present work reveals how shade positively regulates GA_1_ accumulation at the level of GA metabolic enzyme gene expression. Notwithstanding the established metabolic module, upstream transcriptional regulators (e.g., GmPIFs) bridging shade perception to GA metabolic genes remain unidentified, precluding a complete signaling chain. Integrating transcriptomic screening with direct protein-DNA interaction assays (e.g., ChIP-qPCR, Y1H) will be essential to close this gap. Additionally, the current GA profiling, focused on GA_1_ and GA_8_, omits biosynthetic intermediates (e.g., GA_12_, GA_53_, GA_20_) that would fully resolve metabolic flux dynamics. Notably, GA_3_ and GA_4_ showed no significant change, consistent with GA_1_-specific responses.

### 3.4. GA_1_ Metabolism as a Promising Target for Shade-Tolerant Soybean Breeding

The development of shade–tolerant crop varieties is essential for the sustainable intensification of intercropping systems [52]. Traditional breeding approaches for shade tolerance have focused primarily on morphological traits such as plant height and stem diameter, but the underlying molecular mechanisms remain largely unexploited. This study identifies GA_1_ metabolism as a key physiological target for regulating plant architecture in shade–tolerant soybean breeding. Precise modulation of the balance between GA biosynthesis and catabolism holds promise for developing shade–tolerant varieties with moderate plant height, appropriate stem diameter, and enhanced lodging resistance [53].

Importantly, the observed genotypic differences in GA_3_ sensitivity have direct implications for genetic dissection and breeding applications. BX10 and BD2, which are extreme divergent parents, have been used to develop an F_9_ recombinant inbred line (RIL) population comprising 168 families [54]. This RIL population has been phenotyped at four experimental sites (Shijiazhuang, Sanming, Huizhou, and Sanya) and is suitable for constructing a high-density genetic map, dissecting the genetic basis of soybean plant architecture, analyzing genotype-by-environment interactions, and mapping quantitative trait loci (QTLs) for key agronomic traits such as plant height and internode length [55,56,57]. By validating the suitability of BX10 and BD2 as extreme parents for QTL mapping, the present study provides a phenotypic basis for further dissecting the molecular mechanisms underlying plant height regulation. Together with the finding that GA_1_ metabolism serves as a physiological target, this genetic resource paves the way for marker-assisted selection or gene editing targeting key nodes in the GA metabolic pathway. Functional validation herein relies solely on exogenous GA_3_ application, which entails potential off-target effects; consequently, genetic confirmation of GA_1_’s core role and the molecular basis of genotypic divergence await CRISPR-based manipulation or pharmacological inhibition in the BX10 × BD2 RIL background. To fully resolve this mechanistic gap, subsequent studies will further integrate transcriptomic profiling with QTL mapping using this RIL population to dissect the molecular regulatory network underlying genotypic differences in GA sensitivity.

Building on the findings of this study, future research can be expanded in three key directions. First, elucidate the upstream molecular mechanism by which photoreceptors (phytochromes and cryptochromes) regulate GA metabolic reprogramming, and identify key transcription factors (e.g., GmPIFs) that directly modulate GA metabolic gene expression. Second, investigate the dynamic crosstalk between gibberellin and other phytohormones such as auxin under field shading conditions, to construct a comprehensive hormonal regulatory network for soybean shade avoidance. Third, evaluate whether targeted modification of GA metabolism can improve soybean lodging resistance and grain yield in commercial maize-soybean intercropping systems, to promote the translation of basic research findings into agricultural applications.

## 4. Materials and Methods

### 4.1. Experimental Design

#### 4.1.1. Field Experiments

Field experiments were conducted over two consecutive years (2024 and 2025) at the 5th Experimental Field of Xikou Base, Nanping Institute of Agricultural Sciences (118°14′ E, 27°32′). In 2024, soybean cultivar Huaxia 3 and maize cultivar Minshuangse 6 were sown in August and harvested between October and November; in 2025, soybean cultivar Huachun 6 and maize cultivar Minshuangse 6 were sown in April and harvested between June and July. Soybean seeds were inoculated with rhizobium inoculant before sowing in both years without additional chemical fertilizer application. Maize received a basal application of 2250 kg/hm^2^ organic fertilizer, providing 208.6 kg/hm^2^ N, 105.8 kg/hm^2^ P_2_O_5_, and 164.0 kg/hm^2^ K_2_O, all applied uniformly before sowing.

The experiment employed a randomized complete block design with 4 replicates per treatment. Each replicate plot consisted of 3 planting units, with maize rows spaced 40 cm apart, soybean rows 30 cm apart, and a 30 cm gap between maize and soybean rows. Planting densities were set at 3500 plants/acre for maize and 13,200 plants/acre for soybean. Regular field management practices were implemented, including manual weeding and pest control, and plant growth parameters were measured periodically after sowing according to experimental protocols.

#### 4.1.2. Greenhouse Experiments

To functionally verify the role of gibberellin in shade–induced soybean plant architecture establishment, three soybean genotypes, Williams 82, BD2, and BX10 [58], were used in this study. Seven days after seed germination, seedlings were transplanted into 13 L plastic containers and fixed with disposable KT plates with planting holes, with their bases stabilized using sponges. Oxygen stones connected to aeration tubes were placed in the nutrient solution, which was aerated for 5 min every 15 min; the solution was replaced every 2–3 days, and the pH was maintained at 5.8–6.0. Foliar spraying with 10 μM GA_3_ solution was applied as the treatment, while spraying with clear water served as the control. Agronomic traits including plant height, internode length, and stem diameter were measured 20 days after sowing.

#### 4.1.3. Artificial Shading Treatments

In another greenhouse experiment, soybean cultivar Williams 82 was selected as the test material. Black polyethylene shading cloth was hung approximately 2 m above the plant canopy to adjust the photosynthetic photon flux density (PPFD) of soybean to four gradients: 300, 210, 120, and 50 μmol·m^−2^·s^−1^. Notably, the PPFD values of 210 and 120 μmol·m^−2^·s^−1^ were consistent with those measured at 8:00 a.m. and 4:00 p.m. in the field under maize–soybean intercropping shading conditions, respectively. After 15 days of shading treatment, plant height and hypocotyl length of soybean were measured.

### 4.2. Measurement of Photosynthetic Photon Flux Density (PPFD) in Field-Grown Soybeans

Photosynthetically active radiation (PPFD) was measured using a portable photosynthetically active radiometer (LI–COR LI–190R, spectral response range 400–700 nm, accuracy ±5 μmol·m^−2^·s^−1^).This high-precision instrument is produced by, LI-COR, a renowned manufacturer based in Lincoln, NE, USA. Before measurement, the instrument was calibrated with a standard light source to ensure measurement accuracy. Field measurements were conducted on sunny, cloudless days at 8:00 a.m. and 5:00 p.m., focusing on PPFD values at the top of the soybean canopy. Each measurement point was sampled three times at 1–min intervals, and the average value was taken as the final result. Under indoor artificial lighting conditions, the measurement height of the radiometer was set at the top of the soybean plants to determine PPFD values. During the measurement process, environmental parameters such as air temperature and relative humidity were recorded simultaneously.

### 4.3. Histological Analysis of Soybean Stem

Fresh soybean stem segments were cut into thin slices and fixed in FAA fixative solution at room temperature for 24 h. The fixed samples were then subjected to dehydration and paraffin infiltration. Transverse sections with a thickness of 8 μm were obtained using a rotary microtome, mounted on glass slides, and dried overnight. Before staining, the sections were dewaxed in xylene twice for 15 min each time, followed by rehydration through a graded ethanol series. The sections were stained in 0.5% safranin solution for 1–2 h, then differentiated in 50% ethanol for several seconds until lignified tissues turned red and other tissues remained lightly colored. Subsequently, the sections were stained in 0.1% fast green solution for 30–60 s. After rinsing with water, the sections were dehydrated, cleared in xylene, mounted with neutral balsam, and finally observed under a light microscope [59].

### 4.4. Determination of Gibberellin Content

Fresh plant samples stored at ultra–low temperature were ground into powder using a grinder at a frequency of 30 Hz for 1 min. Under liquid nitrogen environment, 50 mg of the ground powder was weighed, and 10 μL of internal standard mixed working solution (10 ng/mL) was added. Extraction was performed by vortexing with 1 mL of methanol–water–formic acid mixture (volume ratio 15:4:1) for 15 min. Subsequently, the mixture was centrifuged at 12,000 rpm at 4 °C for 10 min, and the supernatant was collected. The supernatant was concentrated to dryness in a concentrator overnight, then reconstituted with 500 μL of 3.5% formic acid aqueous solution and 1 mL of ethyl acetate. After vortexing for 10 min, the mixture was centrifuged at 12,000 rpm at 4 °C for 5 min. 800 μL of the upper ethyl acetate layer was transferred to a brown vial, and 500 μL of ethyl acetate was added to the remaining system for repeated extraction. 500 μL of the upper ethyl acetate layer was collected and combined into the corresponding brown vial. The combined ethyl acetate extract was dried under nitrogen, reconstituted with 200 μL of acetonitrile, and vortexed for 5 min. 10 μL of TEA and 10 μL of BPTAB were added, and the mixture was incubated at 90 °C for 1 h. After drying under nitrogen again, the residue was reconstituted with 100 μL of acetonitrile–water solution (volume ratio 9:1), filtered through a 0.22 μm membrane, and transferred to an injection vial for LC–MS/MS analysis. The data acquisition instrument system mainly included ultra–high performance liquid chromatography and tandem mass spectrometry [43].

### 4.5. RNA Extraction

Total RNA was extracted from plant tissues using TransZol reagent (Cat. No. ET101; TransGen Biotech, Beijing, China). Briefly, 50–100 mg of fresh plant tissue was fully ground into fine powder in liquid nitrogen, and immediately transferred to an RNase–free centrifuge tube containing 1 mL of TransZol reagent. The mixture was vortexed thoroughly to ensure complete mixing of tissue powder and reagent, followed by incubation at room temperature for 5 min to complete lysis. Subsequently, 0.2 mL of chloroform was added to each 1 mL of TransZol reagent, and the mixture was vigorously shaken for 15 s, then incubated at room temperature for 3 min. After centrifugation at 12,000× *g* for 15 min at 4 °C, the upper colorless aqueous phase containing RNA was transferred to a new RNase–free centrifuge tube. An equal volume of pre–chilled isopropanol was added, and the mixture was gently inverted to mix well, followed by incubation at room temperature for 10 min. After centrifugation at 12,000× *g* for 10 min at 4 °C, the supernatant was carefully discarded. The RNA pellet was washed with 1 mL of pre–chilled 75% ethanol (prepared with DEPC–treated water), followed by centrifugation at 8000× *g* for 5 min at 4 °C. The supernatant was discarded, and the washing step was repeated once. The RNA pellet was air–dried at room temperature for 5–10 min, then dissolved in 50–100 μL of RNase–free water. The concentration and purity of RNA were determined using a Nanodrop 2000 spectrophotometer, and samples with an A260/A280 ratio between 1.8 and 2.0 were used for subsequent experiments. This precision instrument is manufactured by Thermo Scientific (a division of Thermo Fisher Scientific), headquartered in Wilmington, DE, USA.

### 4.6. Real-Time Quantitative Reverse Transcription Polymerase Chain Reaction (RT–qPCR)

cDNA was synthesized using 2 μg total RNA with TransScript II One–Step gDNA Removal & cDNA Synthesis SuperMix (AQ311, TransGen, Beijing, China). RT–PCR was performed using TransStart Tip Green qPCR SuperMix (AQ141, TransGen, Beijing, China) on a Roche LightCycler^®^ 480 II Real–Time PCR System, which is manufactured by Roche Diagnostics, a global leader in in vitro diagnostics and life sciences based in Basel, Switzerland. The primers used are listed in Table A1.

### 4.7. Statistical Analysis

Differences between treatments were analyzed using Student’s T–test or one–way analysis of variance (ANOVA) combined with Duncan’s multiple range test. Statistical analyses were performed using SPSS 20.0 software (IBM, Armonk, New York, USA), with a significance threshold set at *p* < 0.05.

## Data Availability

The datasets used and/or analyzed during the current study are available from the corresponding author on reasonable request.

## Appendix A

**Table A1:** Primer information.

Primer Name	Sequences
*GmGA20ox1*–qF	CAAAACACATCCGCAGAAAA
*GmGA20ox1*–qR	GTTGGACTGGTTTGGCATCA
*GmGA3ox1*–qF	GCGTAACGGTGAATAGGACAT
*GmGA3ox1*–qR	CGAGCAACAGAGTGAACCAA
*GmGA2ox–7a*–qF	TGTAAAACCAAACCCTGAT
*GmGA2ox–7a*–qR	AACTCTATGCTCCACACTCTT
*GmGA2ox–7b*–qF	ACGGTCTCATGCCACATACT
*GmGA2ox–7b*–qR	TTAACTGCGATCCATTTGCT
*GmEF1α*–qF	TGCAAAGGAGGCTGCTAACT
*GmEF1α*–qR	CAGCATCACCGTTCTTCAAA
